# Two Cases of Transverse Testicular Ectopia in Consanguineous Boys

**DOI:** 10.1055/s-0038-1667329

**Published:** 2018-12-12

**Authors:** Mohamed Abdelmalak, Saber Waheeb, Ahmed Koraitim, Dina Mahdy, Deena Mustafa Abd ElMigeid

**Affiliations:** 1 Department of Pediatric Surgery, Alexandria, El Shatby, Alexandria, Egypt; 2 Department of Pediatric Surgery, Alexandria University, Alexandria, Egypt; 3 Department of Radiology, Egypt Ministry of Health and Population, Alexandria, Egypt; 4 Department of Pharmacy, Faculty of Pharmacy, Alexandria University, Alexandria, Egypt

**Keywords:** transverse testicular ectopia, testicular pesudoduplication, DW-MRI

## Abstract

Crossed testicular ectopia (CTE)/transverse testicular ectopia (TTE) is a rare condition occurring in only 1 in 4 million male patients, in which both testes migrate toward the same hemiscrotum.

We report on two cases of TTE in first degree cousins (1 + 3 years of age).

Both presented with right nonpalpable testis. On diffusion-weighted magnetic resonance imaging, the right testis was located above the left testis in both patients. Right orchiopexy was performed after passing the right testis through the median raphe of the scrotum followed by ipsilateral left scrotal orchiopexy.

## Introduction

Transverse testicular ectopia (TTE) is a rare anomaly in which both testes descend or migrate through a single inguinal canal into the same hemiscrotum. Often, the diagnosis is made during surgical exploration (65%). 1 2 This condition is usually associated with other abnormalities such as persistent Mullerian duct syndrome (PMDS), true hermaphroditism, inguinal hernia, hypospadias, pseudohermaphroditism, and scrotal anomalies. About 100 cases of TTE have been reported in the literature. 1 However, in most of cases the diagnosis cannot be made preoperatively. We report on two consanguineous children.

## Case Reports

Two first degree cousins presented with a right nonpalpable testis which could not be visualized on pelvic ultrasound (US). Therefore, they were referred for abdominopelvic conventional magnetic resonance imaging (MRI) plus diffusion-weighted imaging.

General physical examination was unremarkable, no other congenital anomalies were found, and no family history for the same condition.

On examination, the right testis was nonpalpable inguinally with an empty right hemiscrotum.

The MRIs were performed with a 1.5-T MRI system (Achiva, Philips Medical Systems, Best, Netherlands) using a body coil under sedation.

### Case 1

Physical examination showed the phenotype of a 3-year-old boy with a normal size penis. The testis was palpable in the left groin and a nonpalpable undescended testis was found on the right side.

**Fig. 1:**
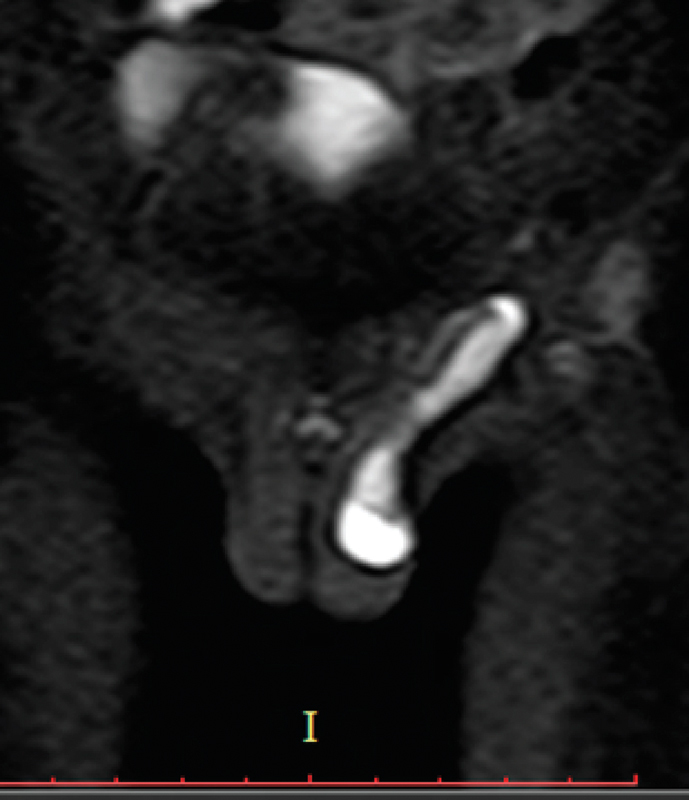
Coronal short tau inversion recovery (STIR) image. Coronal diffusion-weighted image with a b-value of 800. The figure shows the presence of one testis in the left hemiscrotum with hydrocele and another one noted in the left inguinal canal which show hyperintense signal in STIR sequence and restriction in diffusion-weighted image.

On MRI, the left testis was located in the left hemiscrotum, was 1.3 × 0.6 cm in size, and associated with mild hydrocele. Another testis measuring 1.2 × 0.6 cm was noted on the ipsilateral side within the left inguinal canal ( Fig. 1 ).

**Fig. 2:**
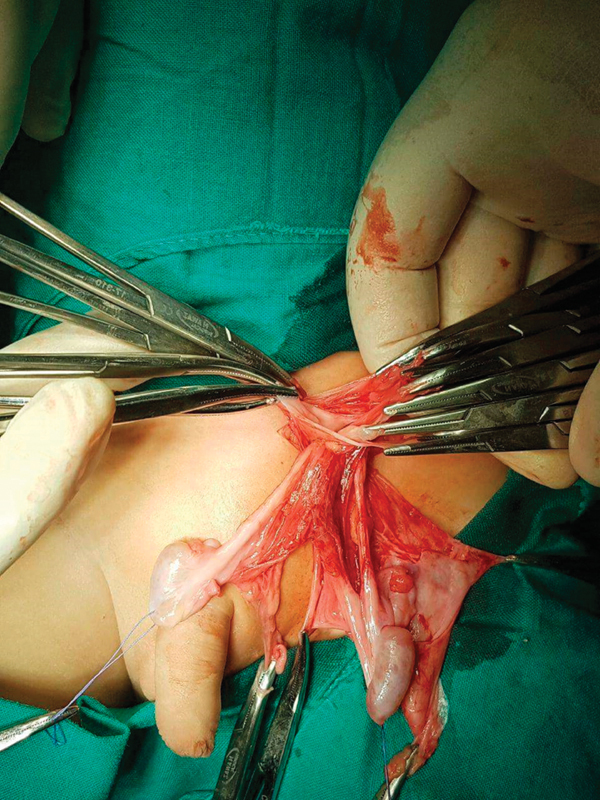
Crossed ectopic testes with separate vas and vessel and a hernia sac.

Exploration of the left groin revealed the presence of both testes within the same side one above the other. They had separate vasa deferentia and testicular vessels for each testis and a common cremasteric muscle and tunical covering. After herniotomy, the ectopic testis was fixed transseptally to the right hemiscrotum ( Fig. 2 ).

### Case 2

Physical examination showed a 1-year-old male with a normal size penis. The testis was felt in the left groin and a nonpalpable undescended testis was found on the right side. In comparison to the right side, the left scrotum was well developed.

**Fig. 3:**
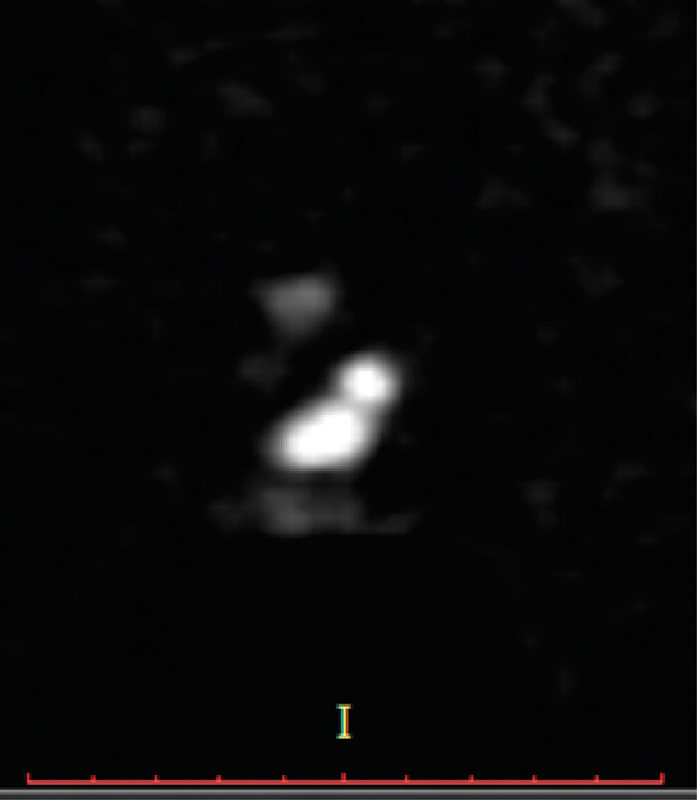
Coronal short tau inversion recovery (STIR) image. Coronal diffusion-weighted image with a b-value of 800. The figure shows the presence of one testis in the left hemiscrotum with hydrocele and another one noted in the left scrotal neck which shows hyperintense signal in STIR sequence and restriction in diffusion-weighted image.

On MRI, the left testis was located in the left hemiscrotum, was 1.2 × 0.6 cm in size, and associated with mild hydrocele. Another testis measuring 1.3 × 0.6 cm was noted at the same side just above the first one near the neck of the scrotum ( Fig. 3a and b ). At this point, the diagnosis of left side TTE was obvious. There was no evidence of inguinal hernia or Mullerian duct structures. Left groin exploration showed a testis over the other near the scrotal neck. Both cords were released followed by bilateral orchidopexy in a subdartos pouch after passing one of them through the median raphe onto the right hemiscrotum.

The suspected diagnosis of a right undescended testis was changed to a left-sided TTE.

**Fig. 4:**
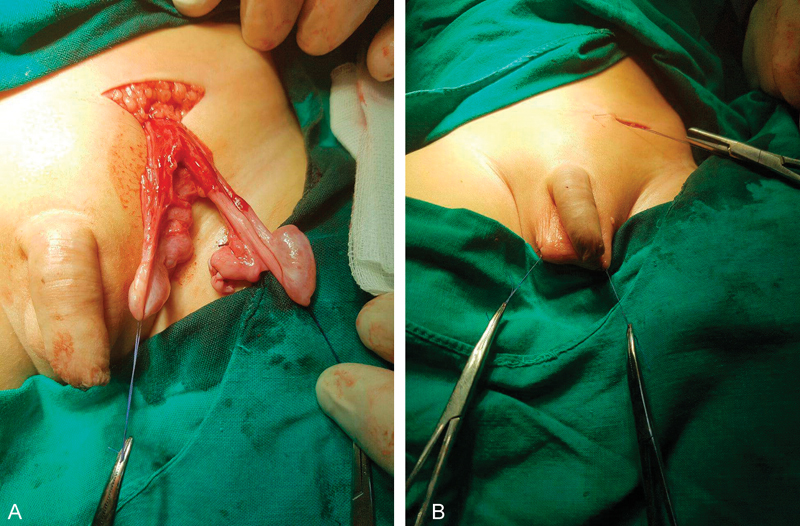
( **A** ) Intraoperative finding of two cords. ( **B** ) Subdartos scrotal fixation of both testes.

Left groin exploration showed a testis over the other near the scrotal neck. Both having common coverings. Both cords were released followed by bilateral orchiopexy using a subdartos pouch after passing one of them through the median raphe onto the right hemiscrotum ( Fig. 4 ).

## Discussion

Ectopic testis may be located in different sites including the superficial inguinal pouch, suprapubic, femoral, perineal, and base of the penis. TTE is a rare anatomical abnormality where both gonads migrate toward the same hemiscrotum. It was first described by Von Lenhossek in 1886. 3 Several theories have been proposed in an attempt to explain the etiology of this rare anomaly. These include the PMDS which may result from the failure of synthesis or release of Mullerian duct inhibitory factor (MIF), the failure of end organs to respond to MIF, or a defect in the timing of the release of MIF. It seems possible that the mechanical effect of the persistent Mullerian duct structures prevents a normal testicular descent. As a result, both testicles descend toward the same hemiscrotum with the appearance of TTE. 2 4 5 6

Despite this variety of theories, the exact etiology of this condition is still not fully understood.

The mean age of presentation is 4 years 1 2 7 and the clinical presentation generally includes an ipsilateral inguinal hernia and a contralateral or sometimes a bilateral cryptorchidism. 5 The diagnosis of TTE can be made preoperatively by clinical examination, use of ultrasonography by an experienced sonographer, and MRI. 8 Although laparoscopy is considered the gold standard, 9 10 MRI may have additional diagnostic value. 11

Our two cases of TTE in first degree cousins are unusual as there was no clinical evidence for an inguinal hernia or other anomalies indicating TTE.

Patients with TTE are at increased risk of malignant transformation, Mullerian remnants, and subsequent malignancy. In one study, a yolk sack tumor developed in a patient who presented with transverse ectopic testes. 12 Wood and Elder showed that the risk of malignancy in undescended testicles is decreased if orchiopexy is performed before the age of 10 to 12 years. 13 Once the diagnosis of TTE is made, orchiopexy is recommended for the preservation of fertility and to prevent malignant transformation. 13 .

Management of testicular ectopia includes either transseptal or extraperitoneal transposition orchiopexy. Our two cases were managed by transseptal orchiopexy with preservation of the vas deferens and testicular vessels.

TTE associated with fused vas deferens is extremely rare. 14 15 16 This condition may hinder the testis from being placed into the scrotum during orchiopexy. In cases of fused vas deferens, a transseptal orchiopexy is recommended.

Our follow-up plan includes a physical examination after 1 month postorchidopexy and then after 6 and 12 months.

## Conclusion

TTE is a rare anomaly with unknown pathogenesis. Hereditary factors may play a role. It should be suspected in patients presenting with impalpable undescended testis on one side and cryptorchidism on the other side.

Also, a thorough clinical examination and laparoscopy imaging such as US and MRI are useful for establishing the preoperative diagnosis of TTE and associated anomalies.

## Notes

New Insights and the Importance for the Pediatric Surgeon Crossed testicular ectopia (CTE)/transverse testicular ectopia (TTE) is a rare condition occurring in only 1 in 4 million male patients so it's needed to be documented, it's presence in consanguineous boys enhance role of hereditary factors in addition to use of DW-MRI to confirm diagnosis and associated anomalies.
